# Impact and experiences of vestibular disorders and psychological distress: Qualitative findings from patients, family members and healthcare professionals

**DOI:** 10.1111/hex.13906

**Published:** 2023-11-01

**Authors:** Laura J. Smith, Wesley Pyke, Rosanna Fowler, Britta Matthes, Emma de Goederen, Shanmuga Surenthiran

**Affiliations:** ^1^ Centre for Preventative Neurology, Wolfson Institute of Population Health Queen Mary University of London London UK; ^2^ School of Psychology, Keynes College University of Kent Kent UK; ^3^ Department for Health University of Bath Bath UK; ^4^ The London Neuro‐otology Centre London UK

**Keywords:** activities of daily living, framework analysis, psychological distress, quality of life, vestibular disorders

## Abstract

**Introduction:**

People with vestibular disorders frequently experience reduced quality of life and challenges with activities of daily living. Anxiety, depression and cognitive problems often co‐present with vestibular disorders and can aggravate symptoms and prolong clinical recovery. We aimed to gain in‐depth insights into the impact of vestibular disorders and the contribution of psychological factors by exploring multistakeholder perspectives.

**Methods:**

Semistructured interviews were conducted between October 2021 and March 2022 with 47 participants in the United Kingdom including: 20 patients (age *M* = 50.45 ± 13.75; 15 females), nine family members (age *M* = 61.0 ± 14.10; four females), and 18 healthcare professionals. Data were analysed using framework analysis.

**Results:**

Vestibular disorders impact diverse aspects of patients' lives including work, household chores, socialising, and relationships with family and friends. Being unable to engage in valued activities or fulfil social roles contributes to feelings of grief and frustration, affecting identity, confidence, and autonomy. Anxiety and low mood contribute to negative thought processes, avoidance, and social withdrawal, which can impede clinical recovery through reduced activity levels, and end engagement with treatment. Coping strategies were thought to help empower patients to self‐manage their symptoms and regain a sense of control, but these require oversight from healthcare providers.

**Conclusions:**

Daily activity limitations, social participation restrictions, and psychological distress can interact to impact quality of life, sense of self, and clinical recovery amongst people with vestibular disorders. Information and resources could aid societal awareness of the impact of vestibular disorders and help patients and families feel understood. An individualised and comprehensive approach that concurrently addresses mental, physical, social, and occupational needs is likely to be beneficial.

**Patient or Public Contribution:**

Two group meetings were held at the beginning and end of the study with a patient and public involvement network formed of people with vestibular disorders and family members. These individuals commented on the study aims, interview schedule, participant recruitment practices, and interpretation of the themes identified. Two core patient members were involved at all stages of the research. These individuals contributed to the formulation of the interview schedule, development and application of the coding scheme, development and interpretation of themes, and preparation of the final manuscript.

## 1 INTRODUCTION

Vestibular disorders encompass a range of conditions that affect the inner ear and brain, causing disruption to an individual's balance, spatial orientation, and sense of motion. Dizziness (general sense of disorientation) and vertigo (erroneous perception of self‐ or object motion) are prevalent within the general population (~30% of adults^1^); a third of these problems are caused by dysfunction of the vestibular system.^2^, ^3^ Symptoms vary across vestibular disorders but include vertigo, unsteadiness, oscillopsia, hearing loss, and tinnitus. The impact of vestibular disorders appears to be considerable. These can increase the risk of clinically significant outcomes such as falls and restricted mobility^4^, ^5^ and contribute to limitations in daily activities including driving, travel, work, and socialising.^6^, ^7^ The quality of life amongst people with vestibular disorders is comparable to other chronic health conditions^8^ and these quality‐of‐life reductions are responsible for heavy societal economic burden of 64,929 USD per vestibular patient across their lifetime.^9^

The psychological consequences of vestibular disorders are also widely acknowledged.^10^, ^11^ People with vestibular disorders frequently experience anxiety, low mood, and panic^12^, ^13^, ^14^; cognitive problems with spatial cognition, attention, and executive function are also common.^15^, ^16^ Alterations to vestibulocortical networks involving brain regions implicated in memory, attention, and emotion regulation are thought to underlie this association.^11^, ^17^, ^18^, ^19^ In addition to secondary difficulties with compensating and adjusting to living with a vestibular disorder.^20^

Importantly, psychological factors can instigate and aggravate dizziness and prolong the impact of vestibular disorders, with complex vicious circles of interaction at play.^21^ Anxiety and depression have been linked to poorer clinical recovery, increased perception of symptom severity, and a higher likelihood of vestibular disorder recurrence.^22^, ^23^ Cognitive deficits can also hinder driving and employment, leading to functional impairments.^24^, ^25^ Psychological factors are, therefore, intrinsically linked with quality of life, with implications for clinical care and daily activities. The coronavirus disease 2019 (Covid‐19) pandemic has brought to the forefront the impact of psychological distress in vestibular disorders. Evidence suggests that although Covid‐19 can induce vestibular symptoms as a consequence of the infection,^26^ psychological distress following acute infection and during social distancing restrictions appears to precipitate and exacerbate vestibular symptoms, particularly lightheadedness, and nonspecific dizziness.^27^, ^28^

Thus far, mostly quantitative studies have been conducted to shed light on the impact of vestibular disorders and experiences of psychological aspects.^15^, ^16^, ^19^, ^29^, ^30^ These have applied a range of self‐report questionnaires, objective cognitive assessments, and experimental paradigms to understand the prevalence and nature of functional and psychological deficits. Currently, there is a lack of research focusing on the lived experiences and daily life challenges faced by individuals with vestibular disorders and how psychological factors contribute to this. Such perspectives are particularly important given that the vestibular system is different to our other senses. The vestibular organs have a constant resting, baseline firing rate and do not provide a conscious sensation, most of us will therefore not usually be aware of their role unless they malfunction.^31^ Vestibular symptoms are difficult for patients to verbalise, making it difficult for clinicians to reach a diagnosis and contributing to poor societal awareness of vestibular disorders.^32^ A deeper understanding of how vestibular and psychological factors are experienced is required to better understand how daily activities are impacted and develop person‐centred care.

Some qualitative studies have begun to explore the lived experiences of stakeholders in relation to the psychological aspects of vestibular disorders. From the patient perspective, Harun et al.^33^ recruited 16 older adults with age‐related vestibular loss who described experiencing problems with fatigue, depression, anxiety, and cognition. Participants also reported having to reduce or slow down their participation in daily activities (including shopping, walking and travelling) and needing help to complete these. Similarly, interviews with 18 bilateral vestibulopathy patients revealed that cognitive and emotional symptoms were experienced, and participants had adjusted to cope with these (e.g., performing tasks more slowly).^34^ More recently, Nagdee et al.^35^ interviewed 14 people with various vestibular disorders about how this had impacted their work, this encompassed engaging with mental health services to overcome negative feelings and address personal factors.

The perspectives of other important stakeholders have also been sought. Story et al.^36^ explored the lived experiences of 10 significant others of people with ongoing vestibular symptoms, highlighting their journey to understanding and coping with these conditions and the diverse impacts these have on the family unit. Furthermore, Walker et al.^37^ interviewed 10 vestibular physiotherapists about their current practice in assessing and treating vestibular patients with anxiety. Participants emphasised the importance of psychoeducation, building an effective therapeutic relationship and access to training resources.

While offering valuable insights, these studies only focussed on the perspective of one stakeholder group. Conducting qualitative research with a varied sample of stakeholders may offer further insights into the impacts of vestibular disorders and how psychological factors contribute to people's experiences, recognising the varying needs, knowledge, and perceptions of different stakeholders.^38^ In this study, we aim to bridge this gap by applying qualitative methodologies to a diverse group of relevant stakeholders including patients, their family members, and healthcare professionals. By gathering the personal narratives of multiple stakeholders, we seek to gain a comprehensive picture of what is meaningful to people with vestibular disorders and foster a more inclusive and collaborative approach to care provision. We aim to explore (1) how people with vestibular disorders experience psychological distress and understand how this affects wellbeing and daily activities, and (2) how psychological aspects contribute to the impact of vestibular disorders.

## 2 MATERIALS AND METHODS

### 2.1 Design

A qualitative study utilising semistructured interviews formed part of a wider programme of research designed to establish current care practices and develop a pathway for the psychological aspects of vestibular disorders. Ethical approval was obtained from the University of Kent Psychology Ethics Committee (202116332980867285). All participants gave informed consent before data collection.

### 2.2 Participants

Eligible participants were 18 years or older, living in the United Kingdom, able to communicate in English, and provide informed consent. We targeted a broad range of stakeholders with complementary perspectives on the psychological aspects of vestibular disorders including people diagnosed with a vestibular disorder, family members of people with vestibular disorders, and healthcare professionals who provide clinical care for people with vestibular disorders.

People with vestibular disorders and their families were recruited through our patient and public involvement (PPI) networks, vestibular charities (Ménière's Society, Vestibular Disorder Association), peer support groups, and by word of mouth. Healthcare professionals had previously participated in a survey on psychological aspects of vestibular disorders^39^ and consented to a follow‐up interview.

### 2.3 Data collection

Interviews were semistructured to allow participants to focus on meaningful issues and to allow for diverse perceptions to be expressed. Semistructured interview schedules were iteratively developed for each stakeholder group (see Supporting Information), based on previous research^38^ and ‘expert reviews’ from researchers, healthcare professionals, and a PPI group comprised of people with lived experience of vestibular disorders. Reviewers provided input on the clarity of the wording used, ordering of questions, relevance of the questions in relation to study objectives, and the number of questions. Two core PPI members were also asked to review the interview schedule and to ‘think aloud’ in response to the questions, this allowed us to determine how they understood the questions and whether their understanding aligned with our expectations. Revisions were made to the interview schedules based on the feedback to enhance the validity of the interview schedule.

Schedules were designed to capture experiences of psychological aspects of vestibular disorders; existing care practices relating to psychological aspects; what a pathway for psychological support could look like; and potential barriers and facilitators to implementation. Interview schedules were informed by the International Classification of Functioning, Disability and Health (ICF)^40^ and Situation‐Inputs‐Outputs‐Mechanism‐Outcome model configuration.^41^ The ICF helped describe the impact of vestibular disorders and understand participants' experiences of psychological distress, while the model configuration helped to map a theory of change for the intervention pathway.

To contextualise the data presented, self‐reported demographic information was collected from people with vestibular disorders and family members, and professional characteristics were gathered from healthcare professionals. Interviews were conducted by two researchers (L.J.S. and W.P.) between October 2021 and March 2022 via videoconferencing and were audio‐recorded. Interviews lasted on average 40 min (range: 19–67 min), were professionally transcribed verbatim and anonymised.

### 2.4 Analysis

NVivo (Lumivero, release 1.6.1) was used to facilitate sorting, coding, charting and organisation of transcribed data. We applied framework analysis, a type of thematic analysis, which allows researchers to explore predetermined objectives (i.e., experiences, impact) while remaining open to explore emergent ideas^42^, ^43^ using a matrix‐based method.

Two authors (L.J.S., W.P.) familiarised themselves with the data by listening to any interview recordings for which they were not present, reading all transcripts, and noting initial ideas in a shared document. These notes were used to develop a preliminary set of codes. We piloted the initial coding framework on five transcripts (~10% of the data set) from different stakeholder groups, iteratively refining the framework by including emergent codes, relabelling codes, and removing duplicates.

Next, guidelines were produced for the coding framework (see the Supporting Information for coding framework). All authors and PPI advisers reviewed and tested out the coding framework with discrepancies resolved through discussion.^43^ The framework was then applied to each transcript by highlighting sections of text and assigning this a code. All transcripts were double‐coded by L.J.S. and W.P. ‘Other’ categories were used to respond to emergent ideas and any disagreements were resolved through discussions with the wider team.

Data were charted using framework matrices to describe participants' experiences and facilitate comparisons across the data set. Initial themes were identified to explain meaning across the data set by L.J.S., W.P. and R.F. These initial themes were then discussed by the wider research team, before being presented to PPI groups alongside example quotes for member checking and feedback to enhance the credibility of the findings.^44^

### 2.5 Rigour

Quality was determined using the guidelines provided by Yardley's evaluative characteristics for good qualitative research.^45^ In particular, the researchers engaged in ongoing discussion, critical reflection, and development of the codes and themes, which included patient partners. Meanwhile, we document all stages of the research process, ensure the confidentiality of participants, and report the findings based on the SRQR Reporting Checklist for Qualitative Data.^46^

## 3 RESULTS

Forty‐seven participants were interviewed. Two potential participants initially expressed an interest in participating but were then unable to do so due to other commitments, or stopped communicating without giving a reason. Table 1 presents demographic data for people with vestibular disorders and their families (a), and professional characteristics of clinicians including how the service they work for is funded and their role (b).

**Table 1:** Participant information: (a) Patient and family member demographics and (b) clinician service type and role.

(a) Patient and family member demographics		
	Patients with VD	Family members
	*N* = 20	*N* = 9
Age		
*M* (SD)	50.45 (13.75)	61.0 (14.10)
Gender		
Female	15 (75%)	4 (44.4%)
Male	5 (25%)	5 (55.6%)
Ethnicity		
White	20 (100%)	9 (100%)
Primary diagnosis^a^		
Ménière's	10 (50%)	
Vestibular migraine	2 (10%)	
Labyrinthitis	3 (15%)	
Bilateral vestibular failure	2 (10%)	
BPPV	1 (5%)	
Acoustic neuroma	1 (5%)	
Perilymph fistula	1 (5%)	
Years since diagnosis		
*M* (SD)	8.0 (6.71)	

(b) Professional characteristics	
	Professional, *N* = 18
Funding	
NHS	10 (55.6%)
Private	5 (27.8%)
Charitable organisation	2 (11.1%)
NHS/private	1 (5.6%)
Role	
Clinical scientist/researcher	4 (22.2%)
Neurologist	2 (11.1%)
Physiotherapist	2 (11.1%)
Neuro‐otologist	2 (11.1%)
Audiologist	2 (11.1%)
Clinical psychologist	1 (5.6%)
Audiovestibular physician	1 (5.6%)
Counsellor	1 (5.6%)
Physiotherapist	1 (5.6%)
Administrator and group liaison officer	1 (5.6%)
GP	1 (5.6%)

*Note*: One participant identified as a family member and a Family 33, quotes from this individual are indicated within Section 3.

Abbreviations: BPPV, benign paroxysmal positional vertigo; GP, general practitioner; NHS, National Health Service; VD, vestibular disorder.

^a^ Eight patients had received multiple diagnoses. The primary diagnosis is indicated in the table.

**Figure 1:**
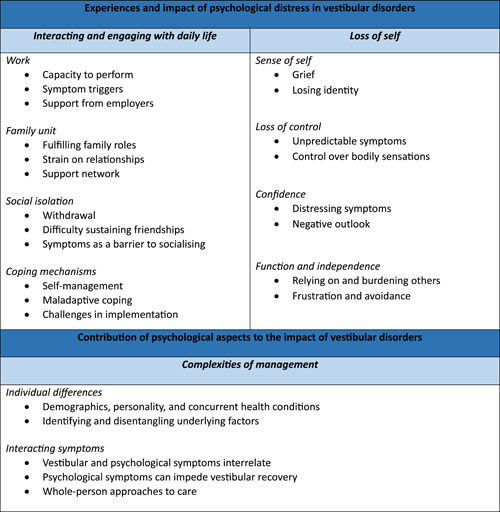
Overview of results.

Results are described below, grouped according to our research questions (see Figure 1). The themes *interacting engaging with daily life* and *loss of self* address the experiences and impact of psychological distress amongst people with vestibular disorders. *Complexities of management* addresses how psychological aspects contribute to the impact of vestibular disorders. Themes are described below with illustrative excerpts extracted from interview transcripts. Where multiple participant groups endorsed a subtheme the term ‘stakeholders’ is used, otherwise the relevant group is specified (e.g., clinicians).

### 3.1 Experiences and impact of psychological distress in vestibular disorders

#### 3.1.1 Interacting and engaging with daily life

Stakeholders described how vestibular disorders affect different facets of daily life. Four subthemes were identified: *work, family and home, social isolation*, and *coping mechanisms*.

Vestibular disorders were described as reducing participants' capacity to complete *work*‐related tasks: ‘It's become increasingly more difficult to work in admin with a vestibular disorder over the years because of screen work’ (Clinician 24). Administration and computer‐based tasks were challenged by vestibular and cognitive symptoms including, visual disturbances, difficulty concentrating, and tinnitus.

He was having attacks at work and having to be sent home, so it was partly physical, but it was partly psychological as well … he was an engineer, what he did had to be right by the millimetre and he was getting unable to concentrate. (Family 37)

Artificial lighting, background noise, and busy environments within the workplace and during the journey to work could trigger symptoms. The opportunity to work from home had helped some people with vestibular disorders to cope with their symptoms and remain in work.

So, I was working from home in the end because I couldn't go out, couldn't stay in work, because every time I'd be there it would be like the lighting would affect me, I'd feel sick. Then all of a sudden, I'd have a drop attack without any warning. (Patient 41)

Busy workloads, fast‐paced workflows, and stress compounded difficulties at work. For some, this led to the onset of an attack at work or an exacerbation of symptoms, presenting setbacks to recovery.

I'd be working long hours, subject to call‐out, fairly public figurehead type role, so sudden attacks of dizziness really impacted on my confidence … I'd quite often get woken up in the middle of the night to go out to something and of course, a lack of sleep or stress can be a trigger, so there was a kind of a cycle really. (Patient 14)

People with vestibular disorders and their families described making decisions to medically retire or change to a less demanding role. Some had concerns for the future and the financial implications of not being able to work to their full capacity.

She's at the point now where she's considering moving, getting another job and I don't know how that will work either because she's got a diagnosis now and unless she gets that completely under control, starting a brand‐new job when you're not in control of these symptoms wouldn't be great. So that is the biggest worry I think for her and for all of us. (Family 46)

They also shared experiences with employers. Some employers had been supportive and put meaningful accommodations in place. People with vestibular disorders particularly valued being able to take a step back when they felt their symptoms worsening or needed a break, but also described not wanting to let others down. Others described employers being unsympathetic or lacking understanding of vestibular disorders and their impact.

I'd been off for eighteen months and trying to agree a phased return, my doctor had written to work saying ‘I think she should be able to work out for herself when she's had enough’, and work said ‘no, we're not having that, she's coming back on a timetable.’ (Patient 12)

Stakeholders also discussed the impact of vestibular disorders on the *family unit* and home environment. Trying to avoid triggering symptoms and managing wellbeing and energy reserves often led to difficulties engaging in valued activities and household chores. People with vestibular disorders and their family members described how this affected their family roles (including parent, partner, breadwinner, and looking after elderly parents).

And then obviously my son suffers because I can't stand him walking around if he's talking to me, I've got nothing left in the tank to kind of follow him, you know … so I know I can get short with him ‘please stand still, stop moving.’ (Patient 5)

Importantly, stakeholders recognised that family relationships can provide a consistent source of support. This encompassed support to carry out daily tasks and caring for an individual when their vestibular symptoms are heightened.

I have to rely on my husband even when we go to certain places … ‘You're 100% in charge of the kids and I need you to lead me through this crowd' or through this area’. (Patient 1)

Family relationships also provided a source of emotional support to cope with distressing symptoms and the impact of vestibular disorders. People with vestibular disorders and their family members felt it was important to listen to and validate an individual's concerns.

The main thing [is], making sure that you're always there for her, whatever she needs. Working at it as a team although, you know, I don't suffer from it but putting myself in her shoes. (Family 35)

People with vestibular disorders and their family members also shared their experiences of the strain vestibular disorders place on family relationships: ‘I think it basically put pressure on my partner … that caused us to break up, I think I just had a complete change of my personality’ (Patient 34). People with vestibular disorders also discussed how the onset of their condition had impacted important life decisions around family planning and moving in with a partner.

I think it made me assess, like, because I live alone … like should I be trying to move in with my partner. (Patient 6)

Family members found it distressing to witness their loved ones in a debilitated state, particularly during an acute attack. They also discussed the challenges of having to put aspects of their lives on hold to care for them or be ‘on call’ in case of a vestibular attack.

When they're ill like this and you're their mum, they just become helpless again like a baby … like do other people feel guilty because they don't always want to go and do these things [supporting during an attack], but it has to be done, you know, it kind of messes with your head a bit. (Family 46)

Anxiety, depression, and irritability experienced by people with vestibular disorders also led to tensions within relationships, particularly if the individual was unaware of how their psychological wellbeing had changed.

I didn't get the impression that he knew what he was doing, and he was being vicious, he just couldn't cope with how he was feeling … we were in a circular situation, I felt resentful because of the way I perceived that he was treating me, which made him worse, which made me worse, which made him worse. (Family 37)

Stakeholders also described how vestibular disorders can contribute to *social isolation* and becoming withdrawn from the outside world. Anxiety and avoidance further contributed to social withdrawal and becoming more sedentary:

If they're feeling unsteady and dizzy, they're more likely to stay at home and not do things and then end up not interactively seeing people. (Clinician 30)

Several people with vestibular disorders felt unable to sustain relationships and friendships due to a lack of awareness about their condition and how it impacts them. This sometimes led to the breakdown of friendships and a reluctance to share how they are feeling with others.

There are some people who don't believe you with this condition and they don't understand so we've lost a lot of friends along the way. And the friendships I do have, like I can't offer anything to them because they never know how I'm going to feel. (Patient 1)

Socialising was also impacted by a perceived lack of control over vestibular symptoms, which meant cancelling engagements and feeling unable to fulfil reciprocal needs within friendships.

The effect on my social life and feeling like I can't commit to something because I don't know until like literally before I'm supposed to leave if I can come and do that. (Patient 6)

People with vestibular disorders described how vestibular symptoms including hearing loss, tinnitus, and dizziness made social environments challenging. Negative experiences meant they were likely to avoid these situations going forward.

You get an invitation for an event where there's a group of people and I have been having to decline these a lot because there's likely to be a lot of noise because by its very nature there's a lot of people, because of the hearing loss I won't actually pick up a quarter of what's being said because it's just a big cloud of noise. (Patient 32)

Stakeholders described practical *coping mechanisms* applied to self‐manage the symptoms of vestibular disorders. Stakeholders described a range of positive strategies, such as pacing, relaxation techniques, and exercise to aid coping and enhance wellbeing. Some felt adopting a positive and proactive approach gave them a sense of control over their vestibular disorder.

I was doing things like mindfulness and different exercises, and I was also trying to start a support group, I was trying to do things for myself basically, because otherwise what would you do, just wait? (Patient 9)

Some acknowledged maladaptive techniques, such as the use of alcohol, which resulted from feelings of stress, desperation, and lack of support.

To be honest I was just mental—well basically I reached out to alcohol as my relaxant from anxiety because I didn't understand how to get help. (Patient 10)

Clinicians emphasised the importance of practical tools to help empower patients, encourage self‐management, and re‐enable engagement with valued aspects of daily living.

It's not just kind of ‘here are your balance exercises and that's what you need to work on’, it's also going through the aspects of pacing yourself so you're not overdoing it one day and then completely wiped out the next. (Clinician 28)

However, family members recognised that people with vestibular disorders sometimes found such resources hard to use in practice, especially when facing particularly severe vestibular symptoms.

We did a lot of meditation … he knows that it's one of the ways that you can destress, but when he's bad he won't do it, so it's like just when you need it, he's sort of unwilling to partake in it. (Family 37)

#### 3.1.2 Loss of self

Psychological wellbeing was impacted by who people feel they were before the onset of a vestibular disorder and what they feel they have consequently lost. Four subthemes were identified: *sense of self, loss of control, confidence*, and *function and independence*.

Stakeholders described how vestibular disorders can impact on one's *sense of self*. No longer being able to perform or participate in valued activities that were once undertaken with ease resulted in feelings of grief amongst people with vestibular disorders, impacting upon their sense of ‘who you are, the self‐concept’ (Clinician 24).

You can feel like a totally different person and there can be a lot of grief and loss around things that you could do before that you can't do, coupled with the fear and anxiety. (Family 46)

Perceived *loss of control* was also commonly discussed by stakeholders. Some described how vestibular symptoms could reduce the sense of ownership and control over the body resulting in anxiety, fear, and low mood.

I still know what it was like, this panic, that I was losing bodily self‐control effectively, and my world was shrinking. (Family 33)

Stakeholders spoke about the unpredictable nature of vestibular symptoms and not being able to anticipate the onset of a vestibular episode. A lack of perceived control over symptoms consequently impacted other aspects of daily living, limiting their ability to move forward.

They don't necessarily have control of their symptoms; they don't know when it's going to come on. A lot of them aren't very internalised, that they can figure out what triggers it. It's kind of that lack of control that spirals into this avoidant behaviour, avoiding social activities, then going into this anxiety and depression kind of circle. (Clinician 22)

Stakeholders described how disabling vestibular symptoms (particularly unpredictable attacks and unsteadiness) reduce people's *confidence* in their ability to undertake daily activities and induce anxiety and low mood:

You can withdraw and kind of lose confidence and so end up, your world becomes smaller and you're less confident. People can become confined to the house and not feel confident about going out. (Clinician 24)

Confidence was also impacted by a resurgence of vestibular symptoms or setbacks to their treatment. Over time, this led to negative expectations of poor outcomes which could further limit recovery through a self‐fulfilling prophecy.

It was immediately after the setback, and I thought I'd lost two months' worth of progress and I put a lot of effort into getting there and suddenly it was just wiped out. I think it got rid of the optimism as I still feel that I could have it all wiped out overnight again. (Patient 43)

Moreover, a lack of autonomy was emphasised, with stakeholders describing how *functioning and independence* are impacted by vestibular disorders: ‘The psychological issues for me are about the feeling that I'm losing my independence’ (*Patient 6*). Patients and family members outlined the assistance required from others to complete everyday tasks:

I'm a very independent person, and this is not me. I feel like my husband's turned into my carer just now, because I live in the sticks, I can't even walk to the local shop. It's too far. And now I can't even jump in the car and go to the local town, because I'm not safe to drive. (Patient 23)

Some also expressed concerns about going out alone or leaving an individual alone at home in case they become unwell or require help. Coping with less independence often induced frustration amongst people with vestibular disorders, who described ‘feeling like you're a burden on people’ (Patient 6).

Yeah, frustration for me, my husband's exceptionally patient and he doesn't seem to be at all fazed by any of it and if I can't do anything and he has to step in last minute … He doesn't seem to mind one little bit, but I really do. (Patient 5)

### 3.2 How psychological factors contribute to the impact of vestibular disorders

#### 3.2.1 Complexities of management

Stakeholders described factors that they perceived to be relevant for understanding and treating an individual's clinical presentation. Two subthemes were identified: *individual differences* and *interacting symptoms*.

Stakeholders described the influence of *individual differences* such as age, gender, and pre/comorbid health conditions on vestibular and psychological symptomology, which meant vestibular disorders impacted individuals differently.

I'm going into and through menopause, I mean, there's such an overlap mentally with that, that it's hard to tease out how much of the irritability, brain fog, forgetfulness, and whatever else, how much do I blame my vestibular condition for it or how much do I blame menopause for that? (Patient 32)

Stakeholders acknowledged that when a patient's difficulties are ‘multifactorial with other elements of health worries going on’ (Clinician 17), then this can complicate the diagnosis and management of vestibular disorders. Disentangling these aspects was thought to be important for providing appropriate care.

A vestibular condition doesn't go away, and it changes with age, it changes with them presenting with different health conditions, it's always there. So, they need that kind of [individualised] support. (Clinician 17)

*Interacting* vestibular and psychological symptoms were thought to complicate the management of vestibular disorders. Several clinicians recognised that cognitive symptoms, including brain fog, could limit functioning and compound the impact of vestibular disorders. Higher levels of anxiety and avoidance were also thought to reduce engagement with rehabilitation strategies if patients are fearful of provoking further symptoms during exercises and activities.

When it's recognised that they've perhaps got high anxiety levels and their emotions are high. Their behaviour maybe is a barrier so … until this person is sort of more open to support or more open to strategies or is feeling more comfortable and less anxious, it's very tricky. (Clinician 15)

Some people with vestibular disorders also acknowledged that their psychological and vestibular symptoms ‘are very closely interlinked’ (Patient 2) and recognised that factors such as stress, brain fog, and fatigue could trigger or exacerbate their vestibular symptoms.

Stress really affects the way that I feel as well. It can make my Ménière's worse. By that I can feel dizzy and more likely to have a drop attack. (Patient 41)

Stakeholders thought a holistic approach to treatment could help address complex interactions between vestibular and psychological symptoms ‘I think especially with dizziness because it's so intertwined with other things, you need to look at the big picture as much as possible’ (Clinician 22). Stakeholders advocated adopting a whole‐person approach, rather than compartmentalising mental and physical symptoms:

It is so closely intertwined, it is not that I can say ‘oh, these are my emotional issues, and these are my physical problems’, because I mean I'm one human being. (Patient 2)

## 4 DISCUSSION

This is the first qualitative study to explore patient, family member, and healthcare provider perspectives on the impacts of vestibular disorders and how psychological factors contribute to this. Many aspects of daily life were impacted including work, household chores, socialising, and the family unit. People with vestibular disorders and their family members emphasised the far‐reaching consequences of vestibular disorders. Not being able to engage in valued activities or fulfil social roles contributed to feelings of grief and frustration, affecting patients' sense of self and autonomy. Anxiety, panic, and low mood contributed to negative thought processes, avoidant behaviours, and social withdrawal, which in turn could impede clinical recovery by limiting activities and reducing engagement with treatment. Stakeholders recognised the potential of lifestyle changes and coping strategies to help empower people with vestibular disorders to self‐manage their symptoms, gain a sense of control, and aid mental wellbeing. However, family members thought patients would need support to implement these, particularly when symptoms are heightened.

Consistent with previous quantitative and qualitative literature, our findings highlight impaired functioning in people with vestibular disorders that impacts quality of life. Like our study, vestibular disorders have been demonstrated to impact employment, travel, driving, social, and family life.^7^, ^33^, ^35^, ^47^, ^48^ While all stakeholders were aware of the diverse impacts on everyday living, patients and family members described significant changes to their life trajectory, suggesting more could be done to aid societal and clinician understanding of the personal and social consequences of vestibular disorders. Our findings contrast with some questionnaire‐based research where social life and relationships^49^ and recreation and home management^50^ were not associated with vertigo and dizziness. It is possible that these domains were related to other factors not controlled for within these studies (e.g., hearing loss, personality traits, other health‐related factors), alternatively the qualitative approach adopted here may have allowed stakeholders to share their personal experiences of sensitive topics.^51^ For example, several stakeholders described the strain placed on personal relationships with some breaking down, and others changing their roles within relationships.

People with vestibular disorders experienced changes to their sense of self, encompassing loss of identity, confidence, independence, and autonomy. Altered sense of self is well‐documented within chronic health conditions.^52^, ^53^, ^54^ Charmaz^55^ proposed that a previously held healthy identity is replaced by an illness identity that includes physical impairments, emotional reactions, and cognitive constructions of the illness.^56^ This is likely to be especially relevant in vestibular disorders since cortical representation of vestibular information is thought to be important for the sense of self and defining the boundary between the self and external world.^57^ In a previous qualitative study with eight persistent postural‐perceptual dizziness patients, Sezier et al.^58^ identified changes in self‐identity caused by vestibular disorders. Participants spoke about feeling like a different person compared with who they were before the onset of their condition becoming less confident, independent, and spontaneous. This was described as influencing personal relationships, socialising, and inducing concerns about the future. Our findings align with Sezier et al.,^58^ but also demonstrate that these identity changes are acknowledged by family members and healthcare professionals, and are experienced by patients with other vestibular diagnoses. Moreover, they highlight grief relating to sense of self and how adjustment to vestibular disorders is affected by psychological factors (including anxiety, low mood, frustration).

Psychiatric disturbances often co‐present with vestibular disorders^12^ and the neural,^17^, ^30^ psychosomatic^59^, and somatopsychic^60^ origins of these are widely discussed within the literature. In line with previous research,^22^, ^23^ here anxiety and depression were thought to modulate confidence, coping, and clinical recovery. Our participants described how psychological factors were intrinsically linked with vestibular symptoms and daily activities; clinicians particularly recognised the barriers these pose to clinical treatment, a finding previously reported by Walker et al.^37^ This implies that further support for psychological aspects of vestibular disorders is required. Our findings align with Acceptance and Commitment Therapy (ACT) models, which help people to grieve, accept discomfort, and increase engagement in activities that meet their personal values.^61^ There is growing evidence that ACT reduces psychological distress and improves psychological flexibility (including not taking negative thoughts literally, mindfulness) in other chronic neurological conditions^62^, ^63^, ^64^ and amongst people with tinnitus.^65^, ^66^, ^67^ Future research could explore the utility of ACT models in chronic vestibular disorders and any adaptions required. Stakeholders also thought an integrated approach to care would be valuable, whereby physical and psychological aspects are addressed concurrently. Recently, combined approaches delivering cognitive behavioural therapy and vestibular rehabilitation concurrently were found to reduce dizziness handicap, psychological distress, and avoidance amongst patients with persistent dizziness.^68^, ^69^ Our findings lend support to this model and suggest that further roll‐out would be worthwhile, as well as future research investigating the longer term impacts of these interventions on daily activities, social engagement and clinical recovery.

Self‐management interventions were also described as an important therapeutic component. Our findings align with previous studies showing coping strategies can help patients to manage their lifestyle and gain a sense of control over their symptoms (e.g., identifying triggers, having a backup plan for unpredictable attacks).^27^, ^70^, ^71^ Our findings also brought another dimension to this discourse by highlighting barriers to self‐management implementation. Family members in our study thought that patients could have difficulties implementing strategies when their symptoms are severe. Our stakeholders also acknowledged that a negative outlook could be a potential barrier. This aligns with previous research showing that self‐help interventions for dizziness appear optimal when delivered in collaboration with healthcare providers to ensure strategies are adaptive and appropriately paced.^72^, ^73^ Moreover, Franklin et al.^74^ highlight the importance of a comprehensive review covering medical, social, occupational, and psychological needs so that information and self‐management strategies can be tailored towards patients' needs and daily activities through a collaborative process. Further research is needed to explore how this can be feasibly delivered within the constraints of healthcare systems and how to tailor support towards relevant individual differences in the context of vestibular disorders (including sociodemographic factors, concurrent health conditions, and personality traits).

Our study and others show that vestibular disorders contribute to restrictions in participation and autonomy, with the general functional status of people with vestibular disorders rated similarly to that of patients with other chronic illnesses including kidney disease and rheumatic disorders.^50^ However, unlike other chronic illnesses, vestibular disorders are poorly understood, underrecognized, and patients do not typically receive support for psychosocial aspects within routine care.^32^, ^39^ Accurate information provision resources could help validate patients' experiences and improve public awareness of the impacts of vestibular disorders if shared with workplaces, family members, and friends. Sharing resources with other healthcare professionals could also be a first step towards facilitating their understanding and involvement in vestibular disorders (e.g., workplace occupational health, occupational therapists, social prescribers). Tailored resources could also be developed to support family members and address the strains placed on relationships and the family unit as identified here. Future research could also develop and evaluate family‐centred support or peer support groups for family members to address their wellbeing needs.^36^

### 4.1 Strengths and limitations

We drew upon a wide range of experiences, including patients (various vestibular aetiologies, at a range of time points postdiagnosis), family members, and healthcare professionals across different healthcare settings and disciplines. These diverse perspectives have enriched our understanding of the impact of vestibular disorders and the contribution of psychological factors. Nonetheless, other healthcare professionals might have added further nuance to our understanding, including occupational therapists addressing activities of daily living through adaptations and coping strategies.

We recruited patients and family members using an opportunistic sample from our PPI network, promotion through vestibular charities, peer support groups, and word of mouth. This, like in most qualitative research, is not (meant to be) a representative sample. Our patient sample was predominantly white middle‐aged females. Although this is in accordance with reported prevalence rates for vestibular disorders,^7^, ^75^ sampling a broader demographic may offer further beneficial insights into the impact of vestibular disorders on psychological distress, sense of self, family dynamics, and occupational aspects. Moreover, since this project was exploring psychological aspects of vestibular disorders, it is possible that those who took part already had an interest in the topic and perspectives of those less aware/interested in the issue were missed. Interviews were also conducted remotely, and this could be prohibitive for some people with vestibular disorders due to unfamiliarity with digital tools or communication problems. We attempted to allay this concern by offering interviews over the phone or via video conferencing and sending study information and videoconferencing guidance ahead of the interview. Patients were also told they could invite a family member to their interview to provide support.

### 4.2 Conclusions

Our findings demonstrate the far‐reaching impact of vestibular disorders on diverse aspects of daily living and how psychological factors compound this. Information provision, training opportunities, and freely available resources could aid societal and clinician awareness of vestibular disorders and help people with vestibular disorders and their family members feel understood. Current support for vestibular disorders is medically focused, a person‐centred holistic approach that considers physical, mental, social, and occupational needs is likely to be beneficial. Self‐management could be a means of delivering this, though it requires input from healthcare providers and a collaborative approach to tailor support to the individual. Early identification and treatment of psychological distress will help reduce the impact of vestibular disorders on the patient and their family unit and facilitate clinical recovery, ultimately reducing the considerable socioeconomic impact of vestibular disorders. Further research to develop effective psychological interventions for vestibular disorders is therefore highly justified.

## AUTHOR CONTRIBUTIONS

Laura J. Smith and Shanmuga Surenthiran contributed to the study conception and design. Laura J. Smith and Wesley Pyke conducted the interviews and data were analysed by Laura J. Smith, Wesley Pyke and Rosanna Fowler. All authors were involved in the interpretation of results. Laura J. Smith and Wesley Pyke drafted the manuscript and Shanmuga Surenthiran, Britta Matthes and Emma de Goederen provided feedback. All authors read and approved the final manuscript.

## CONFLICT OF INTEREST STATEMENT

The authors declare no conflict of interest.

## ETHICS STATEMENT

The study was approved by the University of Kent Psychology Ethics Committee (202116332980867285). Participants provided informed consent before participation and provided verbal consent at the beginning of each interview.

## Supporting information

Supporting information.

## Data Availability

Anonymised excerpts of the transcripts relevant to the study themes will be made available on the Open Science Framework data repository, along with the coding framework, interview schedules and information on the refinement of study themes.
