# Supplementary material for: Impact and experiences of vestibular disorders and psychological distress: Qualitative findings from patients, family members and healthcare professionals
Source: Health Expect. 2023 Nov 1;27(1):e13906. doi: 10.1111/hex.13906 (PMC10757133; doi:10.1111/hex.13906)
Supplement: Supplementary file 1 — Supporting information. [file HEX-27-e13906-s001.docx]

**Supplementary materials**

***Semi-structured interview schedules***

**Semi-structured stakeholder interview schedule – people with vestibular disorders**

**Opening questions**

- Can you tell me about your vestibular condition?

**Psychological symptoms and impact on daily life**

- Do you experience psychological symptoms? Can you tell me what symptoms you experience?
- How do psychological symptoms affect your daily life?
- Do you perceive these psychological symptoms to be related/connected to your vestibular disorder?
  If yes, in what way?

**Current support for psychological symptoms**

- Have you discussed psychological symptoms with someone from your vestibular care team?
- Have you received any support for psychological symptoms within your vestibular care?
- Do you currently implement any strategies to manage or cope with psychological symptoms?
- How comfortable do you feel discussing psychological symptoms with someone from your vestibular care team?
- How important do you think it is to address psychological symptoms in care for vestibular disorders?

**Specific questions**
**Please refer to example assessment and intervention tools.**

Assessment

- Which psychological symptoms are most important to capture?
- Who do you feel comfortable with, in terms of:

1. The person delivering or recommending the completion of the assessment?
2. The person giving you the results of the assessment?

Management/Support

- Who do you feel comfortable with, in terms of:

1. The person recommending the completion of the management/support programme?

2. The person delivering the management/support programme?

- What are your comments about the intervention examples? What’s most relevant? What’s missing? What’s not needed?

Online/Remote approaches to delivery

- What kind of things are you comfortable doing online?
- Do you have access to technology and the internet?
- How do you feel about completing an online screening tool or assessment?
- How do you feel about discussing your psychological symptoms with someone online?

**Outcomes** *(impact, output effect)*

- Having talked about some potential pathways to address psychological symptoms, what would you hope could change for you?
- What are the things that matter to people with vestibular disorders?
- What are the things that are important to you now, and in the future?

**Reflections**

- What are the barriers or obstacles to addressing psychological symptoms in vestibular care?
- How can these be overcome?

Do you feel there’s anything that could be done?

- Is there anything else you would like to tell me about?

**Thank you**

**Semi-structured stakeholder interview schedule – Family members**

**Opening questions**

- Demographics.
- Can you tell me about your family member/friend’s vestibular condition?

**Psychological symptoms and impact on daily life**

- Does your family member/friend experience psychological symptoms? Can you tell me what symptoms they experience?
- How do psychological symptoms affect their daily life?
- How do their psychological symptoms affect the family unit/your relationship?
- Do you perceive psychological symptoms to be related/connected to their vestibular disorder?
- Were you surprised to hear that psychological symptoms can occur in vestibular disorders?
- How aware is your family member/friend of their psychological symptoms?

**Current support for psychological symptoms**

- Has your family member/friend received any support for psychological symptoms?
- Have you received any support as someone who cares for or supports someone with a vestibular disorder?
- Do you currently implement any strategies to help your family member/partner manage or cope with psychological symptoms?
- How comfortable is your family member/friend with discussing psychological symptoms?
- How important is it to address psychological symptoms in care for vestibular disorders?

**Specific questions**

Assessment

- Which psychological symptoms are most important to capture?
- Who do you feel comfortable with, in terms of:

1. The person delivering or recommending the completion of the assessment to your family member/friend?
2. The person giving your family member/friend the results of the assessment?

Management/Support

- Who do you feel comfortable with, in terms of:

1. The person recommending the completion of the management/support programme to you family member/friend?

2. The person delivering the management/support programme to your family member/friend?

- What are your comments about the intervention examples? What’s most relevant? What’s missing? What’s not needed?

Online/Remote approaches to delivery

- Can you give me your general impressions of how your family member/friend finds using technology and doing tasks online?
- What support (if any) would your family member/friend require to complete an online assessment or support programme?

**Outcomes** *(impact, output effect)*

- Having talked about some potential pathways to address psychological symptoms, what would you hope could change for you and your family member?
- What are the things that matter to people with vestibular disorders and their family members?

**Reflections**

- What are the barriers or obstacles to addressing psychological symptoms in vestibular care?
- How can these be overcome?
- Do you feel there’s anything that could be done?
- Is there anything else you would like to tell me about?

**Thank you**

## Semi-structured stakeholder interview schedule – Clinicians, Charity Workers, and Commissioners

**Opening questions**

- Demographic information
- How do you distinguish vestibular disorders from other sources of imbalance/dizziness?
- Do you routinely discuss psychological symptoms with patients?
- How comfortable do you feel discussing psychological symptoms with people with vestibular disorders?
- How important is it to address psychological symptoms in care for vestibular disorders?
- Are there any symptoms which you think are particularly relevant to address?
- Do you perceive psychological symptoms to be related/connected to vestibular disorders? In what way?
- In your experience, to what extent do psychological symptoms drive vestibular disorders?
- In your experience, to what extent do vestibular disorders drive psychological symptoms?
- In your experience, to what extent do vestibular disorders hinder recovery from vestibular disorders?
- Do you currently recommend any strategies or resources to patients to help them understand, manage, or cope with psychological symptoms?

**Specific questions** *(how a pathway could work, mechanisms)*

Usual care

- Are psychological symptoms addressed within routine appointments?
- Is there a psychologist or specialist/specific practitioner currently embedded within your service?
- What referral pathways are available to your service?
- How do you decide who to refer to a specialist/specific practitioner? What do you use to guide your decisions?
- Who makes clinical/treatment decisions within the multidisciplinary team (MDT)? What is the dynamic between different team members?
- What happens with the results of any screening or assessments tools administered? What are they used for?

Pathway for psychological symptoms

- How can we make a pathway for psychological symptoms work in the context of how services are organised at the moment?
- What do you already do to help people attending your service to feel comfortable?

Assessment

- What information should be contained in the psychological assessment?
- When would it be most useful to assess psychological symptoms?
- How would you prefer to access the assessment results?
- Who should receive the results?
- Is there a risk that clinicians and/or patients could misuse the psychological assessment results? If yes, how can those risks be mitigated?

Management/Support

- Clinical content of the intervention/management programme: how it should be organised so that it can work?

**Outcomes** *(impact, output effect)*

- Outcomes for assessment and psychological management programme
- Having talked about some potential pathways to address psychological symptoms, what would you hope could change?
- What outcomes are we not tapping?

**Service commissioning**

- Who sets the priorities?
- Who commissions services in your area?
  - What are the sorts of things that are being commissioned?

**Reflections**

- What are the barriers or obstacles to addressing psychological symptoms in vestibular care?
- How can these be overcome?
- Do you feel there’s anything that could be done?
- Is there anything else you would like to tell me about?

**Thank you**

***Coding framework***

| Code | Description |
| --- | --- |
| Impact of condition | |
| 1.1 Dependency | Dependency or reliance on others. |
| 1.2 Occupational and activities of daily living | Ability to carry out daily tasks including work. |
| 1.3 Socialising | Participating in social activities, interacting with others. |
| 1.4 Relationships | Connections or bonds between those close to us (family, partner, friends). |
| 1.5 Short-term impact | Impact one week to the next. Short-term health and recovery. Making plans from one week to the next. |
| 1.6 Long-term impact | Concerns for future. Planning for the future, anticipating what this might look like. Long-term health and recovery. |
| 1.7 Other |  |
| Symptomology | |
| 2.1 Acute attacks | Intermittent symptoms that often onset quickly and are severe in nature. |
| 2.2 Chronic (background) | Symptoms that present constantly or most of the time in the background. The symptoms that are reported between attacks. |
| 2.3 Cognitive problems | Difficulty concentrating, remembering, comprehending, finding words, navigating etc. |
| 2.4 Sensory overload | Visual dominance, motion sickness, discomfort from busy visual environments, increased sensory sensitivity. |
| 2.5 Fatigue | Feeling mentally/physically tired or drained. Sleep disturbances. |
| 2.6 Anxiety | Worried, stressed, apprehensive, panic, avoidance. |
| 2.7 Low mood | Unhappiness, hopelessness, losing interest in the things you used to enjoy. |
| 2.8 Isolation | Loneliness, reduced contact with others, feeling excluded. |
| 2.9 Comorbid and premorbid conditions | Other demographic, medical, neurological, or psychiatric illnesses which are relevant in contextualising the patient’s symptoms. |
| 2.10 Other |  |
| Capturing (detecting/screening) psychological problems | |
| 3.1 Rationale | Being transparent about the purpose of the assessment and the information gained. |
| 3.2 As a discussion tool | Use to facilitate conversations between patient and clinician. |
| 3.3 Timing | When is an appropriate timepoint to administer the assessment within the care pathway? Repeated administrations (follow-up). Time taken to complete and interpret. |
| 3.4 Content | Symptoms and domains covered by the assessment. |
| 3.5 Delivery format | In-person vs. online, completed independently or with support etc. |
| 3.6 Who delivers | Which member of the clinical team (or someone else) does/could approach and carry out the assessment? |
| 3.7 Interpretation | Requirements and preferences in terms of scoring, interpreting, and understanding assessment results. |
| 3.8 Other |  |
| Managing (support/intervention) psychological problems | |
| 4.1 Content | Content covered by the intervention. Relevant topics, domains, and approaches. |
| 4.2 Self-directed | Actions taken by people to recognise, treat and manage their own health. |
| 4.3 Information provision | Providing content or information about a health condition or health service. |
| 4.4 Support for family | Supporting the whole families of people affected by vestibular disorders. |
| 4.5 Delivery format | Administered in-person vs. online, 1-1 or group format etc. |
| 4.6 Who delivers | Which member of the clinical team (or someone else) does/could approach and carry out the intervention? |
| 4.7 Timing | When is an appropriate timepoint to administer the intervention within the care pathway? How many sessions and how long per session? |
| 4.8 Expertise (intervention providers) | Knowledge and skills required to design, manage, and deliver an intervention programme. |
| 4.9 Other |  |
| Operational aspects of care | |
| 5.1 Time constraints | Funds and resources available. Including:  Time constraints on healthcare providers; appointment schedule in the clinic; time pressures to adhere to.  Long waiting times - Waiting times for services related to vestibular disorder and psychological symptoms. |
| 5.2 Complex diagnostic process | Steps or stages to achieving a diagnosis. Different specialties/healthcare providers consulted with. Diagnostic assessments and communicating medical history. Trying to verbalise non-visible dizziness symptoms that are difficult to describe. |
| 5.3 Access to support | Unmet needs relating to availability of support or services for vestibular disorders and psychological symptoms. Local provision. |
| 5.4 Referral pathways | Triage procedures. Pathways, policies, and referral criteria. Direct and indirect referrals – who are referrals accepted from. Basis for referral decisions. |
| 5.5 NHS vs. private | Differences in the NHS vs. private providers to healthcare. Models adopted and the experiences of those receiving them. |
| 5.6 Commissioning | Process and policies for health service planning, prioritising, and purchasing. |
| 5.7 Other |  |
| Values | |
| 6.1 Comprehensive approach | Treatment of the whole-person considering physical, mental, cultural, and social factors. |
| 6.2 Positive outlook | Focusing on good things and adopting an optimistic attitude. As opposed to rumination and dwelling on negatives. |
| 6.3 Mental and physical interconnectedness | Attitudes towards people with condition. Either symptoms are seen as disconnected i.e., this is all in your head, the problem is purely psychological or purely physical; or they are seen as related and interacting i.e., anxiety can exacerbate dizziness and vice versa. |
| 6.4 Shared experience | Impact and value of shared lived experience. For patients identifying with one another and helping someone going through similar experience. Could also encompass families and healthcare providers. |
| 6.5 Empowerment | Giving patients involvement and control over decisions and actions affecting their health. Give something back to the vestibular community. |
| 6.6 Other |  |
| Mechanisms (drivers, explaining how something works) | |
| 7.1 Feeling listened to | Does the patient feel they have been listened to and understood? Conversely do they feel dismissed and ignored? |
| 7.2 Rapport | Building a relationship or connection with a patient/healthcare provider. |
| 7.3 Validation | Empathetic response that communicates that what a patient experiences is accepted as true. |
| 7.4 Recognition amongst healthcare providers | Awareness of vestibular conditions and psychological symptoms and ability to identify and reflect on these. |
| 7.5 Insight amongst patients | Insight into psychological aspects of their condition and willingness to accept/address these. |
| 7.6 Multidisciplinary approach | Bringing together multiple professions to jointly manage care. |
| 7.7 Training and education of healthcare providers | Learning opportunities to foster knowledge, skills, understanding of vestibular conditions and psychological symptoms. |
| 7.8 Early intervention | Providing support early in the pathway. Addressing issues earlier for maximum benefit and to prevent symptoms worsening. |
| 7.9 Person-centred approach | Tailored and focuses on the needs and circumstances of the individual. Individual differences, heterogenous presentation. |
| 7.10 Other |  |
| Outcomes (impact, output, effect) | |
| 8.1 Short term | Changes that support/intervention should bring about. Quicker or shorter-term change or impact e.g., symptoms reduction, improve skill or knowledge base. |
| 8.2 Long term | Changes that support/intervention should bring about. Bigger picture or ultimate changes which may take years to come to play e.g., cost saving, QoL. |
| 8.3 Content - Functionality | Measures that matter to people with vestibular conditions. Focus on daily activities, vocational, social aspects. |
| 8.4 Other |  |
